# Radionuclide ^131^I-labeled albumin-indocyanine green nanoparticles for synergistic combined radio-photothermal therapy of anaplastic thyroid cancer

**DOI:** 10.3389/fonc.2022.889284

**Published:** 2022-07-25

**Authors:** Xuemei Zhang, Ziyu Yan, Zhaowei Meng, Ning Li, Qiang Jia, Yiming Shen, Yanhui Ji

**Affiliations:** Department of Nuclear Medicine, Tianjin Medical University General Hospital, Tianjin, China

**Keywords:** anaplastic thyroid cancer, human serum albumin, indocyanine green, photothermal therapy, radionuclide therapy

## Abstract

**Objectives:**

Anaplastic thyroid cancer (ATC) cells cannot retain the radionuclide iodine 131 (^131^I) for treatment due to the inability to uptake iodine. This study investigated the feasibility of combining radionuclides with photothermal agents in the diagnosis and treatment of ATC.

**Methods:**

^131^I was labeled on human serum albumin (HSA) by the standard chloramine T method. ^131^I-HSA and indocyanine green (ICG) were non-covalently bound by a simple stirring to obtain ^131^I-HSA-ICG nanoparticles. Characterizations were performed *in vitro*. The cytotoxicity and imaging ability were investigated by cell/*in vivo* experiments. The radio-photothermal therapy efficacy of the nanoparticles was evaluated at the cellular and *in vivo* levels.

**Results:**

The synthesized nanoparticles had a suitable size (25–45 nm) and objective biosafety. Under the irradiation of near-IR light, the photothermal conversion efficiency of the nanoparticles could reach 24.25%. *In vivo* fluorescence imaging and single-photon emission CT (SPECT)/CT imaging in small animals confirmed that I-HSA-ICG/^131^I-HSA-ICG nanoparticles could stay in tumor tissues for 4–6 days. Compared with other control groups, ^131^I-HSA-ICG nanoparticles had the most significant ablation effect on tumor cells under the irradiation of an 808-nm laser.

**Conclusions:**

In summary, ^131^I-HSA-ICG nanoparticles could successfully perform dual-modality imaging and treatment of ATC, which provides a new direction for the future treatment of iodine-refractory thyroid cancer.

## Introduction

Thyroid cancer, one of the most common endocrine tumors, accounted for 3.0% of the 19.3 million new cancer cases worldwide in 2020 and 0.4% of the 10 million global cancer deaths (1). In recent years, the prevalence of thyroid cancer had increased year by year, becoming one of the main diseases affecting people’s health (2, 3). Differentiated thyroid cancer (DTC) cells can maintain similar functions as follicular cells, such as iodine absorption and iodization (2). For patients with DTC, surgery combined with radioactive iodine therapy followed by levothyroxine replacement therapy is a recognized treatment procedure. However, some patients showed an initial or gradual loss of iodine uptake capacity due to dysfunction or even loss of sodium iodide symporter expression in the basement membrane (4). Therefore, advanced DTC and anaplastic thyroid cancer (ATC) remain a challenge in clinical management. At present, many studies have made some progress in the development of drugs to inhibit or reverse the dedifferentiation of thyroid cancer and ^131^I treatment resistance, including a variety of MAPK and PI3K pathway target inhibitors and immunosuppressants (4). However, many patients would experience severe side effects within a few months of starting treatment. In severe cases, dose adjustment, drug suspension, or even discontinuation of treatment was required (5); some patients also developed resistance to targeted drugs (6), resulting in ineffective treatment. Therefore, it is necessary to further develop new cancer treatment methods.

Photothermal therapy (PTT) is one of the emerging cancer treatments widely used in the biological field in recent years. Studies had shown that a slight temperature increase could increase the vascular permeability of the tumor, initiate the recruitment of certain biomolecules or cells, and promote the spread of photothermal agents in the tumor (7). Temperature between 40°C and 45°C could enhance the blood flow and oxygenation capacity of the tumor (8). The increase in vascular permeability could be the leakage of the photothermal agent into the tumor tissue. Higher temperatures could induce tumor damage and allow certain biomolecules or cells to enter the tumor (9).

Human serum albumin (HSA) molecules were widely used as nanocarriers due to the following advantages: excellent blood retention activity, non-immunogenicity, biocompatibility, high water solubility, biodegradability, chemical stability, and glomerular filtration (10, 11). HSA molecule contained abundant binding sites and could be physically cross-linked with a variety of small-molecule drugs through hydrophobic interaction, thereby helping to deliver anticancer drugs into the tumor tissue (12). HSA can be loaded with many molecules like chemotherapeutic drugs, functional near-IR (NIR) drugs (13), gold nanoclusters (14), carbon nanotubes (15), and graphene oxide (16). Among them, indocyanine green (ICG), as a NIR cyanine dye approved by the US Food and Drug Administration for clinical use, could be excited by single-wavelength NIR light to generate strong fluorescence for imaging and PTT. The NIR fluorescence effect of ICG was mainly used to guide the sentinel lymph node location during the operation. However, due to the concentration-dependent coagulation, the instability of the aqueous solution, and the rapid diffusion of the injection site, the application of ICG as a surgical marker was limited (17). At the same time, its application in tumor therapy was limited due to its poor photostability under NIR light stimulation, short systemic cycle, and lack of tumor-targeting function (18). ICG had a high affinity for protein (19). ICG non-covalently bound to HSA has higher fluorescence efficiency and stability than free ICG (20). HSA-ICG nanoparticles (NPs) could be further loaded with a variety of functional drugs, such as chemotherapeutic drugs, metal ions, radionuclides, and other two-dimensional materials, thus being successfully used for multimodal tumor imaging and treatment (21).

**Figure 1:**
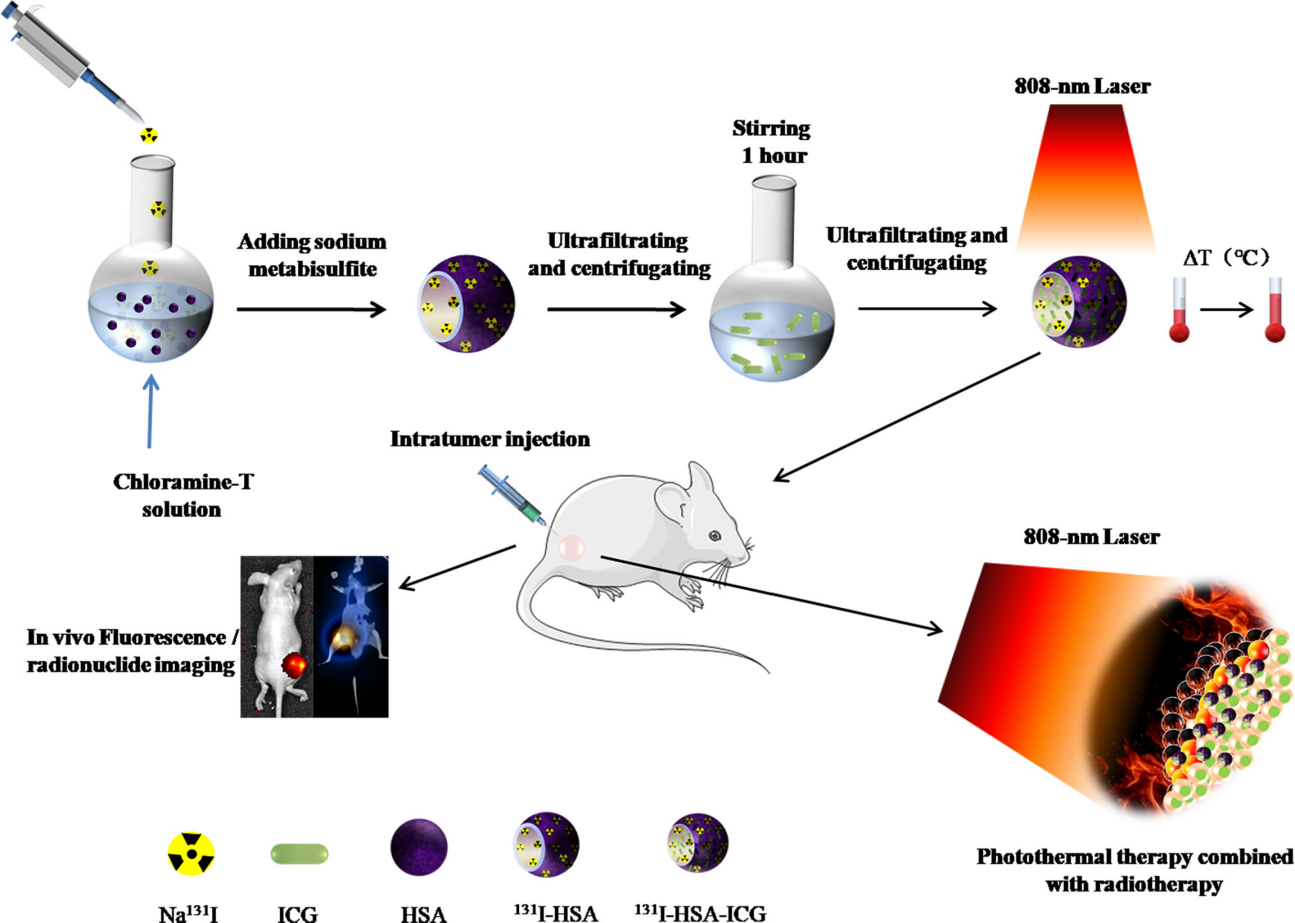
Schematic illustration of synthesized ^131^I-HSA-ICG nanoparticles for bimodal imaging and therapy of anaplastic thyroid cancer. HSA, human serum albumin; ICG, indocyanine green.

Therefore, the goal of this study is to combine ICG with ^131^I to construct an easily retained, safe, and harmless multifunctional NP for locally combined photothermal and radionuclide therapy on iodine-refractory thyroid cancer. The main scheme of our study is shown in **Figure 1**.

## Materials and methods

### Reagents

All of the reagents used in the research were of analytical grade. ICG was bought from Aladdin Biochemical Technology Co., Ltd. (Shanghai, China). HSA was offered by Dingguo Biotechnology Co., Ltd. (Beijing, China). Na^131^I was bought from Tianjin Atom Technology Co., Ltd. (Tianjin, China). Chloramine T was purchased from C&S Biochemical Technology Co., Ltd. (Tianjin, China). Sodium sulfite was purchased from the Beijing Procurement and Supply Station of China Pharmaceutical Company, and sodium iodide (NaI•2H_2_O) was purchased from Bide Pharmaceutical Technology Co., Ltd. (Shanghai, China). Methyl thiazolyl tetrazolium (MTT) was purchased from SDN Company. Dimethyl sulfoxide was purchased from Solebold Technology Co., Ltd. (Beijing, China). Fetal bovine serum (FBS) was purchased from M&C Co., Ltd. Gibco Dulbecco’s modified eagle’s medium, 1640 medium, and pancreas Protease digestion solution [ethylene diamine tetraacetic acid (EDTA)] were purchased from Hai Klonn Biochemical Co., Ltd. (USA). Calcein acetoxymethyl ester (Calcein-AM) and propidium iodide (PI) were purchased from Dongren Chemical Technology Co., Ltd. (Shanghai, China). Physiological saline was purchased from Chinese Otsuka Pharmaceutical Co., Ltd. Five percent chloral hydrate was purchased from the General Hospital of Tianjin Medical University (Tianjin, China). Ultrapure water was purchased from Wahaha Group Co., Ltd. (Hangzhou, China). Ki67 antibody was purchased from FabGennix (Frisco, TX, USA). The urine iodine quantitative detection kit (AR 60T) was purchased from Wuhan Zhongsheng Biochemical Technology Co., Ltd. (Wuhan, China). TLC Scanner was purchased from Hengyide Technology Co., Ltd (Beijing, China).

### Synthesis of HSA-ICG/I-HSA-ICG/^131^I-HSA-ICG nanoparticles

To avoid radioactive damage and contamination, we synthesized non-radioactive I-HSA-ICG NPs for characterization experiments. First, HSA was labeled with iodine by the standard chloramine T method. In 1 ml of phosphate-buffered saline (PBS) (0.01 mol/ml), 20 mg of HSA, 5 mg of chloramine T, and 5 mg of sodium metabisulfite were dissolved separately, and in 1 ml of water, 930 mg of NaI•2H_2_O was dissolved. The HSA, chloramine T, and NaI•2H_2_O solutions were mixed for 1 min. Then 1 ml of sodium metabisulfite solution (5 mg/ml, 1 min) was added to stop the reaction. The reaction solution was ultrafiltered by a centrifuge (TGL-16M, Changsha, China) 3 times (MwCO, 30 kDa; rpm, 6,000 r/min; T, 10 min). Next, according to the molar ratio of HSA : ICG = 1:1, the corresponding concentration of ICG was calculated and added to the mixture. At the same time, another portion of HSA was taken separately and reacted with ICG. The final mixtures were stirred by a magnetic stirrer (Sile Instrument Co. Ltd., Shanghai, China) for 1 h and ultrafiltered 3 times (MwCO, 30 kDa; rpm, 6,000 r/min; T, 10 min). Finally, the synthesized HSA-ICG and I-HSA-ICG NPs were lyophilized and stored away from light. In addition, the NaI•2H_2_O could be replaced with a certain unit of Na^131^I to synthesize ^131^I-HSA-ICG NPs with the same method.

### Characterization of nanoparticles

The hydration diameter and zeta potential of the I-HSA-ICG NPs were measured by a Malvern NP size analyzer (Nano series ZS, UK). The newly obtained I-HSA-ICG NP suspension was transferred to a 200-mesh copper mesh coated with carbon and stained with 2% (weight/volume) phosphotungstic acid. It was dried at room temperature and analyzed by transmission electron microscope (TEM) (Hitachi HT7700, Japan) to obtain the particle size. With pure potassium bromide as the background, the Fourier transform-IR (FT-IR) spectra (400–4,000 cm^−1^) of I-HSA-ICG NPs were recorded by FT-IR spectrometer (Nicol AVATAR-360, USA). The UV–visible–NIR absorption spectra of I-HSA-ICG NPs were measured by UV-3600 plus spectrophotometer (Hitachi, Japan). In addition, static stability observations were performed on ICG and I-HSA-ICG. Both NPs were dissolved in ultrapure water, PB (10 mM, pH = 7.4), FBS, and Gibco 1640 medium. The solutions were allowed to stand for 14 days at room temperature. Photos were taken at different time points (0 h, 0.5 h, 1 h, 2 h, 4 h, 6 h, 12 h, 24 h, 3 days, 5 days, 7 days, and 14 days). During the 14-day observation period, the particle sizes of I-HSA-ICG NPs and the ICG release from I-HSA-ICG NPs in H_2_O and PBS were monitored. In addition, dynamic radioactivity monitoring of ^131^I-HSA-ICG NPs was subjected for 8 days to thin-layer chromatography to evaluate the release of free ^131^I.

Two experimental groups were established. Group 1 included the following solutions with the same concentration of ICG (C_ICG_ = 1 mg/ml): ICG, HSA-ICG NPs, and I-HSA-ICG NPs. Group 2 included I-HSA-ICG NPs with different concentrations. The control group was water. The above solutions were irradiated with 808-nm lasers (2 W/cm^2^, 1 ml, 5 min). During the irradiation, the IR calorific value camera (Filler E75 series, USA) was used to record the calorific value images of each solution, and the temperatures were recorded every 30 s. After irradiation, the I-HSA-ICG solution was left to stand and centrifuged every 30 min. The supernatant was taken, and an equal volume of background solution was added to the stock solution. According to the principle of iodine-catalyzed arsenic–cerium reaction, the iodine release of I-HSA-ICG was monitored within 3.5 h after illumination through the instructions of the urine iodine quantitative detection kit.

An 808-nm laser (2 W/cm^2^) was used to irradiate water (1 ml) and I-HSA-ICG solution (4 mg/ml, 1 ml). The irradiation was stopped after the temperature of the solution no longer increased. The initial ambient temperature was reached naturally. Temperatures were recorded every 30 s during heating and cooling. The light-to-heat conversion efficiency (η) of I-HSA-ICG NPs was calculated by the following formula (22):


$$\eta =\frac{hs\left(T_{max}-T_{surr}\right)-Q_{0}}{I\left(1-10^{-A}\right)}$$

In the formula, *h* is the thermal conductivity and *s* is the surface area of the container. *T_max_* − *T_surr_* represents the maximum temperature change of the I-HSA-ICG NP solution measured at a stable ambient temperature. *Q*_0_ represents the light radiation heat absorbed by the container and the solvent. *I* is the power density of laser radiation. *A* is the absorbance of I-HSA-ICG NPs at 808 nm.

### Cell proliferation

The human undifferentiated thyroid carcinoma cell lines ARO and FRO were kindly provided by Dr. Shunichi Yamashita and Dr. Norisato Mitsutake (Department of Molecular Medicine, Atomic Bomb Disease Institute, Nagasaki University Graduate School of Biomedical Sciences, Nagasaki, Japan). ARO, FRO, and mouse breast cancer cells (4T1 cells) were plated in 96-well plates at a density of 1 × 10^4^ cells/well. Cells were cultured in a constant temperature incubator at 37°C with 5% CO_2_. When the cell density reached 80%–90%, the cells in each group were treated differently. Finally, the cells were treated with MTT, and cell viability was determined by a microplate reader (LabSystems Multiskan MS).

### *In vitro* cytotoxicity of HSA-ICG/I-HSA-ICG nanoparticles

Group 1 formulated HSA-ICG NPs into gradient concentrations (0–3,200 mg/L) and incubated them with FRO cells and 4T1 cells for 24 h. In group 2, HSA-ICG/I-HSA-ICG NPs were formulated into gradient concentrations (0–1,000 mg/L) and incubated with ARO cells for 24 h. Finally, cell viability was obtained by the standard MTT method.

### *In vitro* radioactive lethality of ^131^I-HSA-ICG nanoparticles to cells

ARO cells were seeded in a 96-well plate at a density of 3 × 10^3^ cells/well. On the next day, the cells were treated with Na^131^I and ^131^I-HSA-ICG NPs in different radioactivity gradient counts (radioactivity counts 0, 7.81, 15.63, 31.25, 62.5, 125, 250, and 500 μCi, C_ICG_ = 10 ppm) and co-cultured for 24 h. Cell survival rates were measured by the standard MTT method.

### *In vitro* photothermal lethality of nanoparticles to cells

Different concentrations of I-HSA-ICG/^131^I-HSA-ICG NPs were co-cultured with ARO cells in 96-well plates for 24 h. Each well of different groups was irradiated by an 808-nm laser with different densities for 5 min. The control solution was PBS. The radioactive dose of each well was 500 μCi. Cell survival rates of each group were acquired by the standard MTT method.

### *In vitro* killing of tumor cells by radionuclide therapy combined with photothermal therapy

ARO cells were treated with different methods. Cells in groups 1, 2, and 3 were respectively co-cultured with PBS, ^131^I-HSA (500 μCi/well), and ^131^I-HSA-ICG (500 μCi/well) for 24 h. Cells in groups 4, 5, and 6 were respectively co-cultured with PBS, HSA-ICG, and ^131^I-HSA-ICG (500 μCi/well) for 24 h and then irradiated by an 808-nm laser (2.5 W/cm^2^) for 5 min. Among them, the content of ICG in each group was 11.7 ppm. The cell viability of each group was determined by the standard MTT method. A 1:1 ratio of PI and Calcein-AM mixture was prepared. The above procedures were repeated, and live/dead cell staining of the mixture was carried out after the end of the treatment. Fluorescence images of dead and live cells were acquired by an inverted fluorescence microscope.

### Construction of a tumor-bearing mouse model of anaplastic thyroid cancer

The animals used in this study were male BALB/c nude mice aged 4–5 weeks and weighing 15–18 g (Beijing Huafukang Biotechnology Co., Ltd., China). ARO cells were planted under the skin of the right/left side of the mouse thigh. Mice were treated when subcutaneous tumors had grown to about 6 mm in diameter. One week before treatment, all nude mice in the radionuclide treatment group were given 1% NaI aqueous solution, and 1 ml of 1% NaI aqueous solution was intraperitoneally injected 1 day before treatment. The aim was to block the thyroid glands of nude mice and avoid uptake of radioactive ^131^I during treatment. The animal experiments were conducted in accordance with the Tianjin Medical University Institutional Animal Care and Use Committee guidelines.

### *In vivo* toxicity of I-HSA-ICG nanoparticles

Male Kunming mice (Beijing Huafukang Biotechnology Co., Ltd., China) aged 4–5 weeks were randomly divided into 2 groups, with 5 mice in each group. Group 1 was injected with 100 μl of PBS through the tail vein; group 2 was injected with 100 μl of I-HSA-ICG solution (5 mg/ml) through the tail vein. During the 30-day observation period, the body weight changes of the mice at different time points were recorded. After 30 days, the heart, liver, spleen, lung, and kidney of the mice were removed for H&E staining. The eyeball blood samples of the mice were taken for biochemical examination.

### Evaluation of the effect of ^131^I-HSA-ICG nanoparticles on local tumor retention

The tumor-bearing nude mice were randomly divided into 4 groups with 3 mice in each group. Groups 1 and 2 were injected intratumorally with 100 μl of pure ICG and I-HSA-ICG NPs (C_ICG_ = 100 ppm), respectively. Intravital fluorescence imaging was then performed. Groups 3 and 4 were injected intratumorally with 100 μl of Na^131^I and ^131^I-HSA-ICG NPs (800 μCi/mouse), respectively. Then single-photon emission CT (SPECT)/CT imaging (Discovery 670, General Electric) was performed. By delineating regions of interest on SPECT/CT slices, the radioactivity counts of tumor, liver, kidney, and lung were monitored at different time points within 8 days.

### *In vivo* radionuclide therapy combined with photothermal therapy

Tumor-bearing nude mice were randomly divided into 5 groups with 3 mice in each group. Groups 1 and 2 were injected intratumorally with 100 μl of Na^131^I and ^131^I-HSA-ICG NPs (800 μCi/mouse), respectively. Groups 3, 4, and 5 were injected intratumorally with 100 μl of PBS, HSA-ICG NPs, and ^131^I-HSA-ICG NPs (800 μCi/mouse), respectively, followed by 808-nm laser irradiation for 10 min. The ICG content of all experimental groups was 40 ppm. During the irradiation period, a calorific value camera was used to record and photograph the local temperature increase of the irradiated part. All groups were observed for 17 days after the end of all treatments. Changes in body weight and tumor volume of nude mice at different time points were recorded and photographed. After 17 days, the tumors of the mice were removed and weighed. Six days after treatment, Ki67 antibody immunohistochemical staining was performed on tumor tissues of each group according to the instructions.

### Statistical analysis

The data were all expressed as mean ± SD. The differences between groups were compared by independent-samples t-test or two-tailed t-test. When the *p*-value was less than 0.05, it represented a significant statistical difference (*p* < 0.05).

## Results

### Synthesis and characterization of nanoparticles

**Figure 2:**
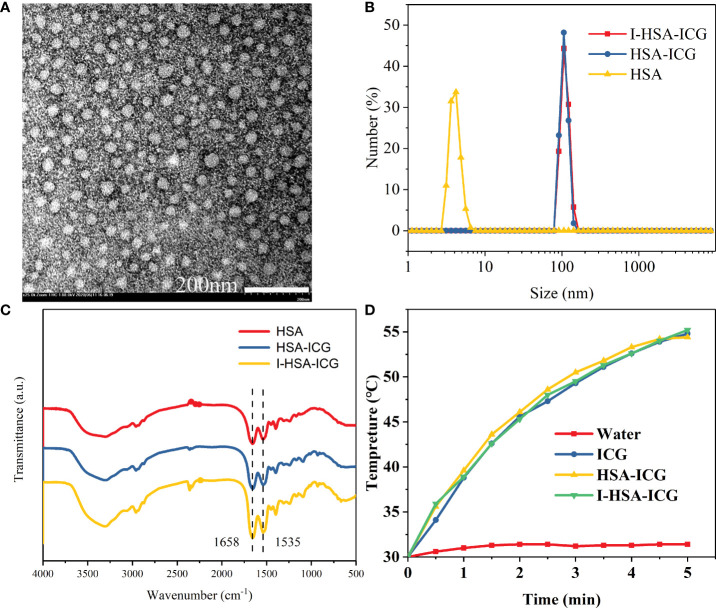
Characterization of I-HSA-ICG nanoparticles. **(A)** TEM image of I-HSA-ICG nanoparticles. Scale bars: 200nm. **(B)** Hydration diameter of HSA, HSA-ICG, and I-HSA-ICG in pure water. **(C)** FT-IR spectra of HSA, HSA-ICG, and I-HSA-ICG. **(D)** The photothermal heating curve after irradiating water and solutions of pure ICG, HSA-ICG, and I-HSA-ICG nanoparticles (C_ICG_ = 0.1 mg/ml) with a power density of 2.0 W/cm^2^ at 808 nm for 5 min. HSA, human serum albumin; ICG, indocyanine green; TEM, transmission electron microscope; FT-IR, Fourier transform-IR.

*In vitro* characterizations were performed to confirm our successful synthesis of the NPs. I-HSA-ICG NPs were spherical with a diameter range of 25–45 nm observed by TEM (**Figure 2A**). The hydraulic diameter distribution range of I-HSA-ICG NPs was 91.3–142 nm (**Figure 2B**), with an average diameter of 115 nm, which was significantly larger than that of the corresponding TEM results. The reason was that dynamic light scattering showed an average hydrodynamic particle size, while the TEM image detected the dehydrated form of the I-HSA-ICG NPs (23). The presence of HSA was determined by measuring the characteristic peaks of the FT-IR spectrum of the I-HSA-ICG NPs. The IR spectrum of the protein showed three amide bands (I, II, and III), of which the amide I band is more sensitive to conformational changes and mainly used for secondary structure analysis. Amide I band appeared in the region of 1,600–1,700 cm^−1^ (mainly caused by C═O stretching vibration). Amide II band appeared between 1,500 and 1,600 cm^−1^ (because CN stretching vibration was accompanied by NH bending mode). The amide III band appeared at 1,200–1,300 cm^−1^ (mainly caused by NH bending mode and CN tensile vibration) (24–27). **Figure 2C** shows the ATR-FTIR spectra of HSA, HSA-ICG, and I-HSA-ICG. The FT-IR spectra of HSA-ICG NPs and I-HSA-ICG NPs both showed two main absorption peaks, which were in the absorption bands of amide I (at 1,655 cm^−1^) and amide II (at 1,535 cm^−1^). Both of the peaks correspond to the characteristic peaks in free HSA. In addition, the zeta potentials of HSA-ICG and I-HSA-ICG NPs at different concentrations in PB buffer and water averaged about −3 and −16 mV (**Supplementary Table 1**), indicating that ICG and I have been successfully encapsulated.

HSA-ICG NPs were synthesized in a molar ratio HSA : ICG = 1:1 and then diluted to a gradient mass concentration to measure its UV–Vis–NIR (**Figure S1A**). A fitting curve of absorbance was drawn at 808 nm (**Figure S1B**). After the molar mass of HSA was fixed and ICG was added with different molar mass ratios, the absorbance at 808 nm was measured by UV–Vis–NIR spectroscopy (**Figure S1C**), and a fitting curve was drawn (**Figure S1D**). According to the two fitting curves and formulas, the ICG content in I-HSA-ICG NPs of a certain quality could be estimated roughly. In addition, pure ICG and I-HSA-ICG NPs were dissolved in different solvents for static stability observation. The particle size of I-HSA-ICG NPs in water stabilized between 98 and 223 nm within 9 days at rest (**Figure S2A**). The release of ICG from I-HSA-ICG in H_2_O and PBS was periodically monitored. The results showed minimal ICG shedding from I-HSA-ICG, even with slight fluctuations at the 72-h time point (**Figure S2B**). The distributions of radioactive counts of ^131^I-HSA-ICG and its released free ^131^I in different solutions were monitored by thin-layer chromatography. It showed that the ^131^I-HSA-ICG radioactivity counts at different time points within 8 days accounted for more than 60% (**Figure S2C**), which indicated less release of ^131^I from ^131^I-HSA-ICG at different time points. After the I-HSA-ICG was irradiated, the iodine content in the supernatant was measured every 30 min. The results showed that although I-HSA-ICG gradually released iodine, the amount of iodine released became less and less over time. Most of the iodine was still on the I-HSA-ICG NPs (**Figure S2D**). During the observation of 14 days, I-HSA-ICG NPs showed a better solubility than pure ICG in various solution media, including water, PB buffer (10 mM, pH = 7.4), FBS, and Gibco 1640 medium. The pure ICG began to settle within 0.5 h after dissolution in FBS. As the observation period extended, the colors of pure ICG in each solution became lighter gradually, which indicated photobleaching of pure ICG (**Figure S2E**). In conclusion, our synthesized I-HSA-ICG NPs had the advantages of suitable size, good NIR absorption region, high stability, and not being easy to decompose.

### Photothermal properties of I-HSA-ICG nanoparticles

The photothermal performance of I-HSA-ICG NPs was evaluated by *in vitro* photothermal heating experiment. During irradiation, except for water, the solutions in the ICG group, the HSA-ICG group, and the I-HSA-ICG group could all be elevated by about 25°C (**Figure 2D**). With the use of the same power density (2 W/cm^2^) to irradiate water and different concentrations of I-HSA-ICG solutions, it was found that an ideal photothermal effect could be achieved when the concentration was 4 mg/ml (**Figure S3A**). In addition, the calorific value image showed that the higher the concentration of I-HSA-ICG NPs, the better the heating effect (**Figure S3B**). The photothermal conversion efficiency (η) of I-HSA-ICG NPs at 808 nm was 24.25% (**Figures S3C, D**), indicating that I-HSA-ICG NPs could be used as an effective photothermal conversion agent to ablate tumors.

### *In vitro* cytotoxicity, photothermal ability, and radioactive killing ability of HSA-ICG/I-HSA-ICG/^131^I-HSA-ICG nanoparticles

**Figure 3:**
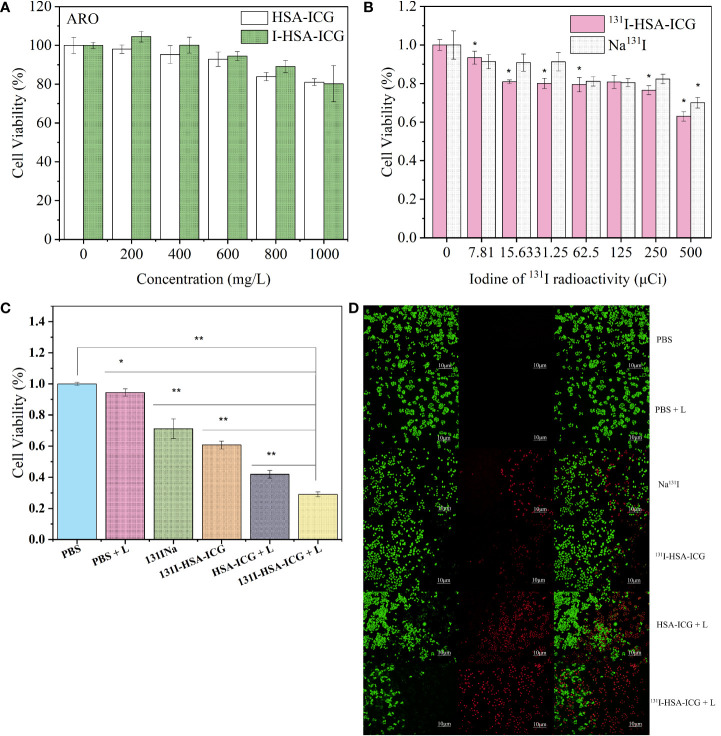
**(A)** Cell viabilities of ARO cells after treatment with different concentrations of HSA-ICG nanoparticles and I-HSA-ICG nanoparticles. **(B)** Cell viabilities of ARO cells after treatment with different radiation doses of Na^131^I and ^131^I-HSA-ICG nanoparticles (C_ICG_ = 10 ppm). **(C)** The survival rates of ARO cells in different treatment groups (C_ICG_ = 10 ppm, 2.5 W/cm^2^). **(D)** Live/dead cell staining pictures of different treatment groups obtained with a fluorescence inverted microscope (C_ICG_ = 10 ppm, 2.5 W/cm^2^). L: 808-nm laser irradiation. HSA, human serum albumin; ICG, indocyanine green *, p < 0.05; **, p < 0.01.

First, the toxicity of HSA-ICG/I-HSA-ICG NPs to tumor cells was evaluated. HSA-ICG/I-HSA-ICG NPs with appropriate concentrations showed no obvious toxicity after incubation with 4T1 cells, FRO cells, and ARO cells, and the cell survival rates were above 70% (**Figure 3A**; **Figure S4A**). ARO cells were used to evaluate the effect of tumor ablation *in vitro*. After ARO cells were incubated with Na^131^I and ^131^I-HSA-ICG for 24 h, cell viabilities were measured by the MTT method. It was found that as the radioactive dose increased, the cell survival rates gradually decreased. The survival rates of 500 μCi/well were about 60% (**Figure 3B**), and there was no significant difference in the lethality of Na^131^I and ^131^I-HSA-ICG in cells.

Next, evaluate the lethality of I-HSA-ICG/^131^I-HSA-ICG NPs on ARO cells under the irradiation of an 808-nm laser. It was found by MTT assay that the cell viability decreased with the increase of drug concentration and laser power density. Under the irradiation of an 808-nm laser at 2.5 W/cm^2^, the survival rates of ARO cells incubated with 1.5 mg/ml of I-HSA-ICG NPs were about 40% (**Figure S4B**). ARO cells were incubated with different concentrations of ^131^I-HSA-ICG NPs. PTT was performed 24 h later with 808-nm laser irradiation at different power densities. When the ICG concentration was 10 ppm, a power density of 2.5 W/cm^2^ could kill most of the ARO cells compared to the control group (**Figure S4C**).

Finally, survival rates of ARO cells in the following different treatment groups were measured: PBS, PBS + light, ^131^I-HSA, ^131^I-HSA-ICG, HSA-ICG + light, and ^131^I-HSA-ICG + light (C_ICG_ = 10 ppm, 2.5 W/cm^2^). As shown in **Figure 3C**, the cell survival rate of the ^131^I-HSA-ICG + light group was about 20%, indicating that the experimental group had a significant therapeutic effect at the cellular level. ARO cells of different treatment groups were carried out using Calcine-AM and PI solution to live/dead cell dye. Green represents living cells, and red represents dead cells. The results confirmed that the ARO cell mortality rate in the photothermal and radioactive treatment group was the highest (**Figure 3D**), which was consistent with the MTT results.

### *In vivo* toxicity of I-HSA-ICG nanoparticles

PBS and I-HSA-ICG NPs were injected into Kunming mice *via* tail vein. All mice showed a steady upward trend in body weight over the 30-day observation period (**Figure S5**). H&E staining of the tissues after 30 days showed that no obvious necrosis, morphological changes, or apoptosis were found in the heart, liver, spleen, lung, and kidney of the mice in each group (**Figure S6**). The biochemical results of eyeball blood showed that there was no significant difference between the PBS group and the I-HSA-ICG NP group, and they were both within the normal range (**Supplementary Table 2**). In conclusion, the I-HSA-ICG NPs have good biocompatibility *in vivo*.

### *In vivo* tumor imaging capabilities of nanoparticles

**Figure 4:**
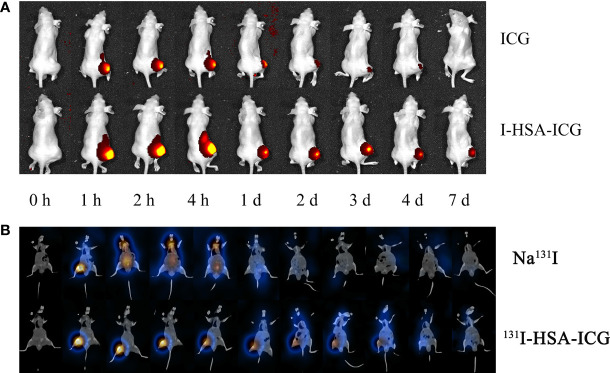
**(A)** *In vivo* fluorescence imaging of pure ICG and I-HSA-ICG at different time points. **(B)** SPECT/CT tomographic images of Na^131^I and ^131^I-HSA-ICG at different time points. ICG, indocyanine green; HSA, human serum albumin; SPECT, single-photon emission CT.

Intratumoral retention of NPs was assessed by performing *in vivo* imaging. *In vivo* fluorescence imaging of small animals showed that I-HSA-ICG NPs could remain in the tumor for more than 7 days, while the ICG in the control group disappear faster (**Figure 4A**). SPECT/CT imaging showed that Na^131^I began to gradually spread to other parts of the body 15 min after injection and was mostly excreted after 24 h. In contrast, ^131^I-HSA-ICG NPs did not begin to spread from the tumor site to other sites until about 24 h after injection and could remain at the tumor site for 4–6 days (**Figure 4B**). The distribution of radioactivity counts in tumor, liver, kidney, and lung of nude mice at different time points was dynamically monitored *in vivo*. The results showed that Na^131^I accumulated in the tumor within 1 h and then spread to the liver, kidney, lung, and other parts continuously. After 24 h, fewer radioactivities were counted in each organ and tumor site of nude mice (**Figure S7A**). It showed that Na^131^I was quickly metabolized mainly by the liver, kidney, and lung. In contrast, ^131^I-HSA-ICG mainly accumulated at the tumor site and persisted until 6–8 days. A few radioactive counts were seen in the liver, kidneys, and lungs, with the liver and kidney being the most prominent (**Figure S7B**). This indicated that ^131^I-HSA-ICG was mainly metabolized by the liver and kidney and a small part was excreted by the lung. Therefore, ^131^I-HSA-ICG had excellent intratumoral retention ability, which could fix ICG and ^131^I at the tumor site for a long time.

### *In vivo* therapy of ^131^I-HSA-ICG nanoparticles

**Figure 5:**
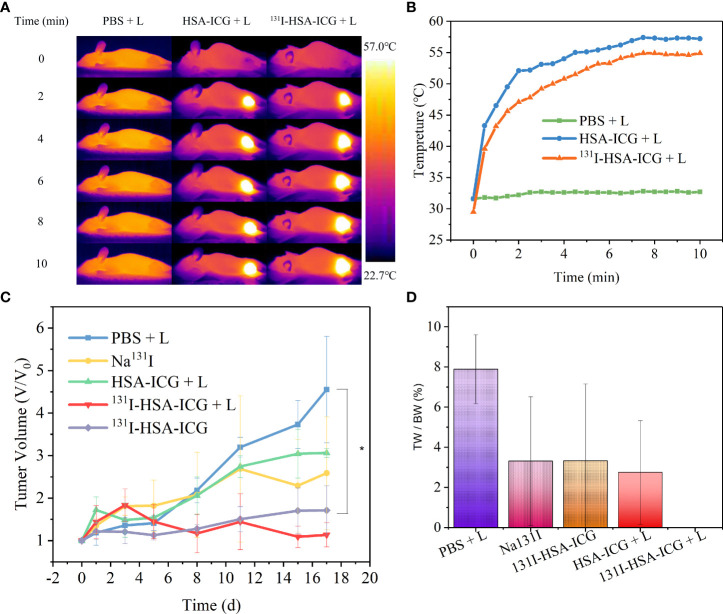
**(A)** Infrared calorific value images of tumor surface temperature changes in nude mice during 808-nm laser irradiation (C_ICG_ = 2 mg/kg, 0.5 W/cm^2^, 5 min). L: 808-nm laser irradiation. **(B)** The tumor surface temperature change curves of nude mice in each group during 808-nm laser irradiation (C_ICG_ = 2 mg/kg, 0.5 W/cm^2^, 5 min). **(C)** The trends of tumor volume changes in nude mice in different treatment groups, expressed as mean ± SD; *, *p* < 0.05. **(D)** The ratio of tumor weight (TW) to body weight (BW) after 17 days in different treatment groups; *, *p* < 0.05. ICG, indocyanine green; HSA, human serum albumin; SPECT, single-photon emission CT.

Tumor-bearing nude mice were randomly divided into groups and received different treatments, including PBS + laser, Na^131^I, ^131^I-HSA-ICG, HSA-ICG + laser, and ^131^I-HSA-ICG + laser. The IR calorific value camera showed that the local tumor surface temperatures of nude mice in the experimental group were significantly higher than in the control group (**Figure 5A**). Both HSA-ICG NPs and ^131^I-HSA-ICG NPs could increase the tumor surface temperature to over 50°C after 10 min of 808-nm laser irradiation. In contrast, the temperature of the control group had almost no significant increase (**Figure 5B**). Nude mice were observed for 17 days after treatments. Tumor tissue samples were taken for immunohistochemical staining with Ki67 antibody on the 6th day of observation. The results showed that the expression of Ki67 in the radioactive ^131^I treatment groups was close to that of the control group but was slightly decreased in the HSA-ICG photothermal treatment group. However, when PTT was combined with radioactive iodine therapy, the expression of Ki67 was significantly reduced (**Figure S8**). The tumor volumes of the nude mice in the HSA-ICG + laser group showed a gradually increasing trend, indicating that PTT had a certain killing effect on tumor cells but could not inhibit tumor recurrence. Compared with the control group, both Na^131^I and ^131^I-HSA-ICG NPs could inhibit tumor growth but not completely ablate tumors. The tumor volume of nude mice in the ^131^I-HSA-ICG + laser group increased slightly in the early observation period, which was considered to be caused by tumor necrosis and edema. Tumor volume was significantly reduced on day 5 after treatment, with little apparent growth thereafter (**Figure 5C**). Photographs of local tumors in nude mice at different time points within 17 days after treatment and the ratios of tumor weight to body weight in nude mice after 17 days also confirmed the above results (**Figure 5D** and **Figure S9**). During the observation, the body weights of nude mice in each group decreased temporarily but gradually became stable (**Figure S10**). These results indicate that the therapeutic effect of PTT combined with radionuclide therapy is the most obvious.

## Discussion

Albumin is the most abundant serum protein of all troponins, with a size of approximately 66.5 kDa (584–590 amino acids) (28) and a circulating half-life of approximately 19 days (29). It interacts with cellular receptors, including the glycol-proteins Gp60, Gp30, and Gp18, and the secreted acidic and cysteine-rich protein, which could be used for specific targeted delivery of NPs (30, 31). In addition to high biocompatibility and non-immunogenicity, HSA has been shown to accumulate and remain in solid tumor tissues due to enhanced permeability and retention effects (29). NPs based on HSA as a carrier had high hydrophilicity and carried a negative charge, which helps to enrich less protein on the surface of NPs after entering the tissues, reducing the damage of protein corona on the pharmacokinetics and physiology of NPs (32). The presence of HSA on the surface of NPs reduced non-specific interactions of serum proteins that might affect the properties and behavior of NPs (31) and helped prolong systemic circulation of NPs after surface coating and overcome uptake by the immune system (28). NPs with preformed albumin protein coronas could inhibit the adsorption of other proteins upon contact, especially opsonins, thereby reducing phagocytosis by macrophages and delaying systemic clearance of NPs (33). Lacroix et al. demonstrated that modification of the dendritic alkyl chains on DNA nanocages exhibited high-affinity binding to HSA and thus significantly prolonged the serum half-life of nanostructures compared to nanostructures without HSA-binding properties (29). Therefore, HSA was a potential candidate in the field of nanomedicine and cancer therapy.

As a natural drug carrier, HSA had the characteristics of low immunogenicity, good biocompatibility, good biodegradability, and long circulation *in vivo*. As a low-toxic and safe clinical dye, ICG had a high affinity for proteins (19) and could non-covalently bind to HSA (20). ^131^I therapy (RAI) was the mainstay of standard treatment for DTC (34). ^131^I induced DNA damage or increased frequency of micronuclei in peripheral blood lymphocytes. However, any possible genetic damage caused by exposure to therapeutic ^131^I might resolve after about 1 year (35, 36). In addition, long-term infertility, miscarriage, and fetal malformation rates did not appear to be increased in women after RAI. Moderate RAI activity also did not affect male fertility and the risk of miscarriage or congenital anomalies in subsequent pregnancies (34). RAI patients were usually advised to avoid contraception within 6 months to 1 year after treatment, which also facilitated the body to repair and renew the most relevant DNA damage caused by IR exposure. Our ^131^I-HSA-ICG NPs combined photothermal and radiotherapy, thus allowing further optimization of radioactive dose and reduction of radiation-induced genotoxicity. Free ^131^I would be excreted quickly after injection, and it could hardly inhibit the growth of tumor cells (37). ^131^I could bind to proteins by electrophonic substitution into aromatic residues. The abundant functional groups on HSA enabled it to covalently bind to compounds such as peptides and radionuclides (38, 39). Therefore, our study used ^131^I to label the abundant tyrosine in HSA to obtain radioactive HSA for radioisotope therapy guided by SPECT imaging. The combination of ^131^I and HSA could significantly increase the uptake and retention of materials in cells (40). HSA loaded with ^131^I or other materials, such as chemotherapeutic drugs and photothermal preparations, could effectively improve the therapeutic effect of tumors by synergizing multiple treatments. Liu et al. (37) used a dimer cRGD-modified HSA nanosystem combined with albendazole and ^131^I to synchronize radiotherapy and chemotherapy for triple-negative breast cancer. The NPs had high aggregation at the tumor radiation site. It could significantly inhibit tumor growth by inhibiting tumor proliferation and promoting cell apoptosis *in vivo*, thereby effectively combining the effects of radiotherapy and chemotherapy. Tian et al. (41) confirmed that HSA-based ^131^I combined with paclitaxel (PTX) could effectively synergize radioactive iodine therapy and chemotherapy in animal tumor models. They found that changing PTX to MnO_2_ could also improve the efficacy (42).

Our study utilized the NIR dye ICG for PTT. HSA-ICG NPs could stay in the tumor site for a long time. Through fluorescence imaging and photoacoustic imaging, it was helpful to guide the surgical resection of the tumor and the photothermal treatment of the tumor cells (43, 44). However, PTT with ICG-HSA had some drawbacks: unnecessary damage to normal tissues led by overheating (45), lower temperature in deep tumors due to limited laser penetration (46), limited dosage of HSA to prevent blood pressure drop and immune response (47), and increased quenching ability of ICG when binding with more HSA (47). In summary, it is necessary to pair HSA-ICG NPs with other treatments to ameliorate the abovementioned defects. In recent years, many multimodal treatment methods based on HSA-ICG NPs have also appeared. Ji et al. (48) combined Chlorin e6 (Ce6) with HSA-ICG NPs for simultaneous photoacoustic and photothermal treatment of prostate cancer. ICG could turn off the phototoxicity of Ce6 and avoid its phototoxicity to normal tissues such as skin and eyes under sunlight. After 808-nm laser irradiation, ICG produced a photothermal effect to kill cancer cells and at the same time turned on the phototoxicity of Ce6 to promote the progress of photoacoustic therapy. Sun et al. (49) used HSA-ICG NPs to modify corolla-like Pd NPs to enhance the NIR absorption capacity, thereby increasing the efficiency of light-to-heat conversion. In addition, loaded chemotherapeutics, such as cisplatin (50) and PTX (51, 52), have also been proven to have significant synergistic therapeutic effects.

Therefore, this study combined ICG and ^131^I *via* HSA to perform synergistic PTT and radionuclide therapy on tumors. ^131^I-HSA-ICG NPs are of suitable size (25–45 nm). The *in vitro* and *in vivo* experiments confirmed that the NPs have objective biological safety and high stability. Compared with pure ICG and Na^131^I, ^131^I-HSA-ICG NPs could be retained in the tumor for a long time, which might be related to the enhanced permeability and retention of NPs (41). ICG highly absorbed light from 650 to 950 nm, which was a relatively transparent window for biological tissues, also known as the treatment window (53, 54). A temperature of 41°C–42°C could expand the blood vessels of the tumor and promote the entry of drugs into the tumor tissue (55), which might be the reason for the accumulation of ^131^I-HSA-ICG NPs in tumor cells. Our ^131^I-HSA-ICG NPs strongly absorbed light at 808 nm and could be heated to over 50°C at non-toxic concentrations. Its photothermal conversion efficiency was 24.25%. Notably, the addition of iodine did not affect the photothermal efficiency. Temperatures above 48°C could cause irreversible damage to cancer cells within minutes (55, 56). Cell experiments confirmed that the radiation damage effect of ^131^I inhibited cell proliferation and growth. Hyperthermia (>50°C) treatment combined with continuous ^131^I radiation could cause significant damage or even death to cancer cells. Compared with ICG, Na^131^I, and HSA-ICG NPs, the tumors in the ^131^I-HSA-ICG NPs treatment group were significantly damaged or even ablated after 808-nm laser irradiation. Therefore, the combination therapy efficacy of radionuclide ^131^I and ICG is significantly better than that of single therapy.

In conclusion, our work combined radionuclide therapy with PTT to construct multimodal imaging and therapy integrated nanomaterial for diagnosis and treatment. ^131^I-HSA-ICG NPs could stay in tumor cells for a long time, which constantly helped in using radioactive radiation to induce cell damage during and after PTT, thereby further enhancing the tumor ablation rate. This method provides an optimization strategy for the combined treatment of HSA, and this platform can be used to further develop other types of nanomaterials to optimize efficacy in the future.

## Data availability statement

The raw data supporting the conclusions of this article will be made available by the authors, without undue reservation.

## Ethics statement

The animal study was reviewed and approved by Experimental Animal Ethics Committee of Tianjin Medical University.

## Author contributions

Conceptualization: ZM. Methodology: XZ and ZM. Software: QJ and YS. Validation: XZ and ZY. Formal analysis: XZ. Investigation: XZ and ZY. Resources: ZM, QJ, YS, and YJ. Data curation: XZ. Writing—original draft preparation: XZ and ZY. Writing—review and editing: ZM. Visualization: XZ. Supervision: ZM and NL. Project administration: ZM. Funding acquisition: ZM, YJ. All authors contributed to the article and approved the submitted version.

## Funding

This study was supported by the China National Natural Science Foundation grant 81571709 and 81971650, the Tianjin Science and Technology Committee Foundation grant 21JCYBJC01820, the Key Project of Tianjin Science and Technology Committee Foundation grant 16JCZDJC34300, and the Project of Tianjin Science and Education Commission grant 20JCQNJC01610. This study was supported by the Tianjin Medical University General Hospital New Century Excellent Talent Program, the Young and Middle-aged Innovative Talent Training Program from Tianjin Education Committee, and the Talent Fostering Program (the 131 Project) from Tianjin Education Committee, Tianjin Human Resources, and Social Security Bureau (awarded to ZM). This study was supported by Tianjin Medical University General Hospital (the Departments of Nuclear Medicine and Radiology).

## Conflict of interest

The authors declare that the research was conducted in the absence of any commercial or financial relationships that could be construed as a potential conflict of interest.

## Publisher’s note

All claims expressed in this article are solely those of the authors and do not necessarily represent those of their affiliated organizations, or those of the publisher, the editors and the reviewers. Any product that may be evaluated in this article, or claim that may be made by its manufacturer, is not guaranteed or endorsed by the publisher.
