# Supplementary material for: Radionuclide 131I-labeled albumin-indocyanine green nanoparticles for synergistic combined radio-photothermal therapy of anaplastic thyroid cancer
Source: Front Oncol. 2022 Jul 25;12:889284. doi: 10.3389/fonc.2022.889284 (PMC9358776; doi:10.3389/fonc.2022.889284)
Supplement: Supplementary file 1 [file DataSheet_1.docx]

Supplementary Material

# Supplementary Figures and Tables

## Supplementary Figures

**
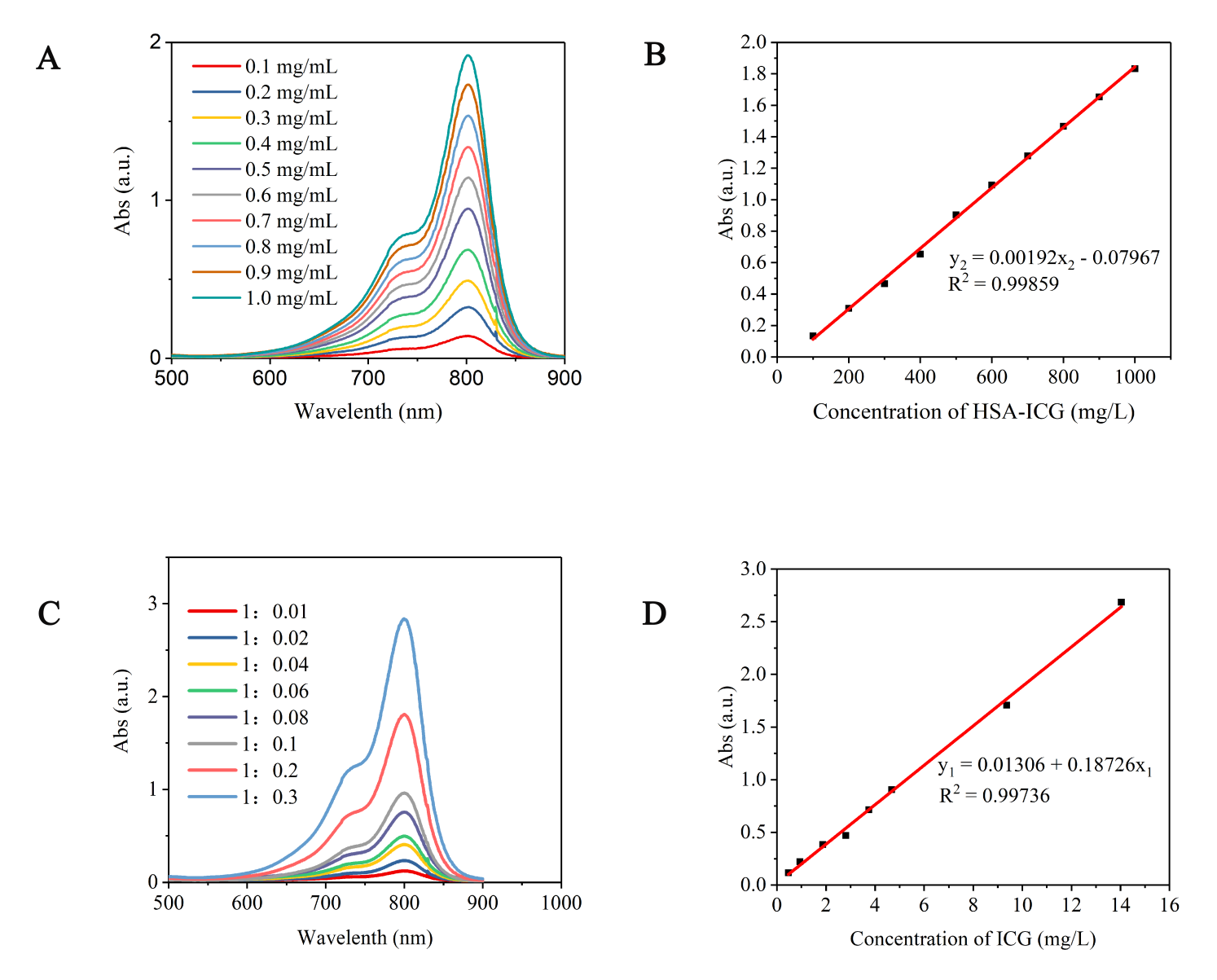
**

**Supplementary Figure 1.** (A) Synthesize HSA-ICG nanoparticles according to different molar mass ratios (HSA: ICG = 1: 0.01, 1: 0.02, 1: 0.04, 1: 0.06, 1: 0.08, 1: 0.1, 1: 0.2, 1: 0.3 ), respectively measure it’s UV-vis-NIR absorption spectrum. (B) Fitting curve of UV-vis-NIR absorption spectrum at 808 nm of HSA-ICG nanoparticles synthesized in different proportions. (C) Synthesize HSA-ICG according to the ratio of 1:1, and measure the UV-vis-NIR absorption spectra of different mass concentrations. (D) Fit curve of UV-vis-NIR absorption spectra of HSA-ICG nanoparticles with different mass concentrations at 808 nm.

**
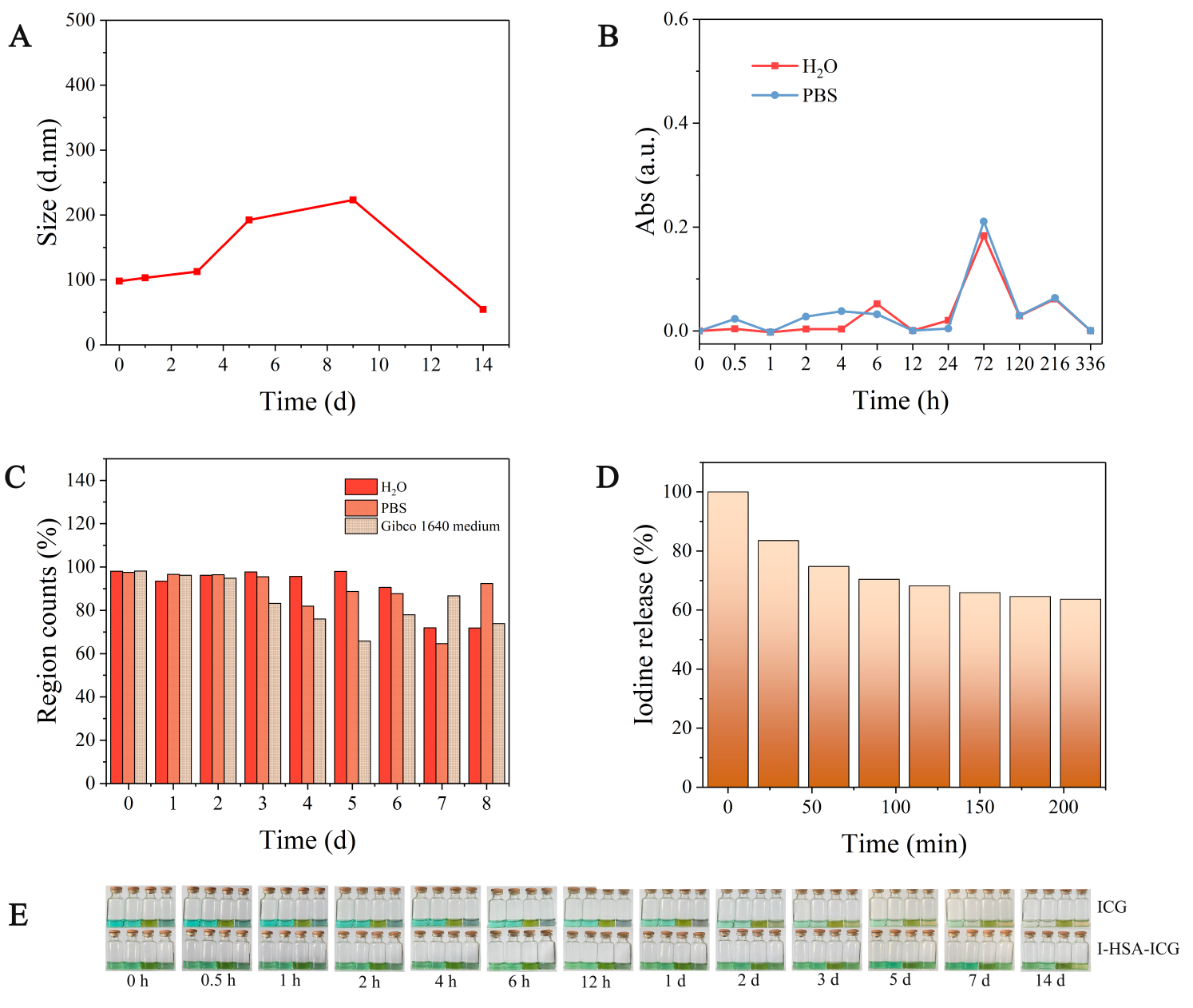
**

**Supplementary Figure 2.** Standing stability and drug release behavior of I-HSA-ICG nanoparticles. (A) Hydrodynamic size monitoring of I-HSA-ICG nanoparticles. (B) The ICG release profile of I-HSA-ICG during 14 days in H_2_O and PBS. (C) The radioactive residual proportion of ^131^I-HSA-ICG in H_2_O, PBS and Gibco 1640 medium at different time points. (D) Iodine residues after NIR laser-induced iodine release from I-HSA-ICG nanoparticles. (E) Photos of ICG and I-HSA-ICG (C_ICG_ = 0.1 mg/mL) dispersed in different media (from left to right: water, PBS, FBS, Gibco 1640 medium) for 14 days.


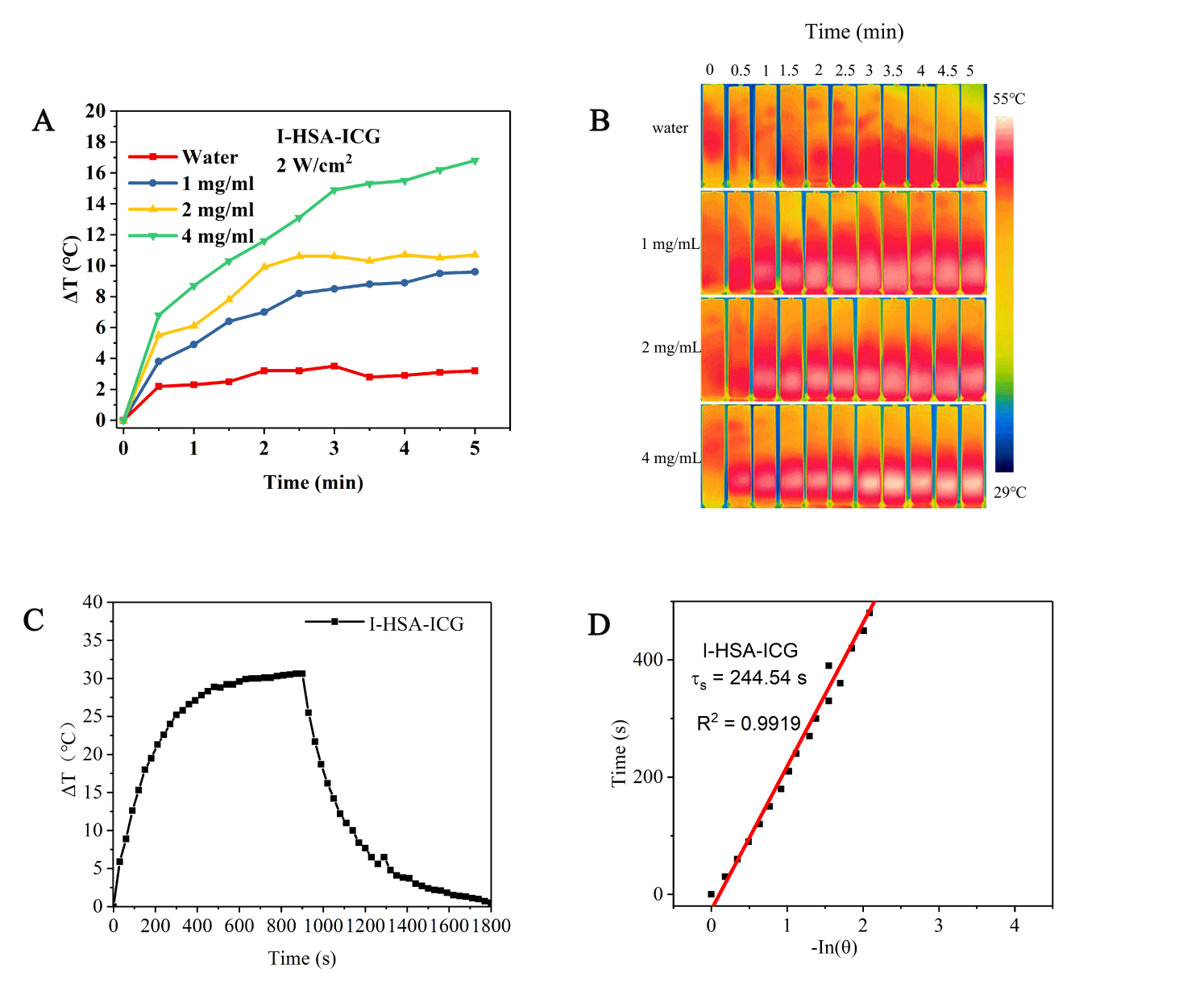


**Supplementary Figure 3.** (A) The photothermal heating curve obtained after irradiating water and different concentrations of I-HSA-ICG nanoparticle solutions (2.0 W/cm^2^, 5 min). (B) The infrared calorific value image obtained after irradiating water and different concentrations of I-HSA-ICG nanoparticle solutions (2.0 W/cm^2^, 5 min). (C) The photothermal heating effect of I-HSA-ICG nanoparticles (C_ICG_ = 0.1 mg/mL) under the irradiation of 808 nm laser with a power density of 2.0 W/cm^2^, the laser was turned off after 15 minutes of continuous illumination. (D) Thermal conduction time constant (In(θ)) of I-HSA-ICG nanoparticles. It was expressed as the negative number of the linear time value after the 900 s cooling period and the natural logarithm of the temperature.


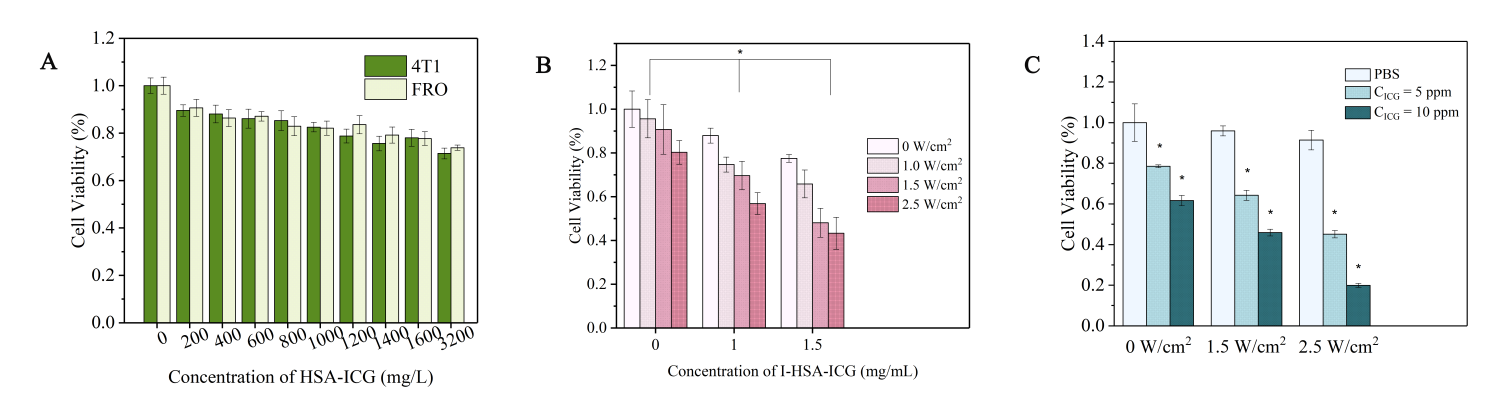


**Supplementary Figure 4.** (A) Cell viabilities of 4T1 and FRO cells after treatment with different concentrations of HSA-ICG nanoparticles. (B) The lethality of I-HSA-ICG nanoparticles on ARO cells was evaluated under the irradiation of 808 lasers with different power densities. The concentrations of I-HSA-ICG nanoparticles were divided into 0 mg/mL, 1 mg/mL, and 1.5 mg/mL. (C) The survival rates of ARO cells after incubation with ^131^I-HSA-ICG nanoparticles containing different concentrations of ICG and then irradiated by 808 nm lasers with different powers.

**
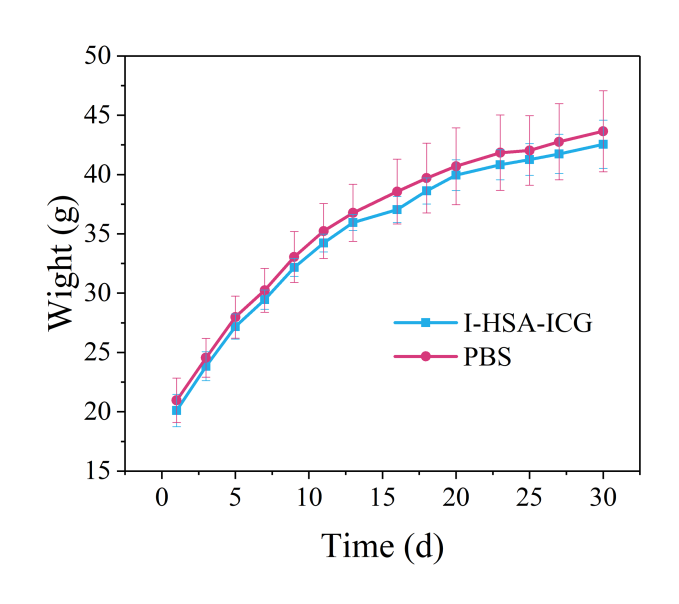
**

**Supplementary Figure 5.** Changes in body weight of mice in each group within 30 days after tail vein injection of PBS and I-HSA-ICG nanoparticles.

**
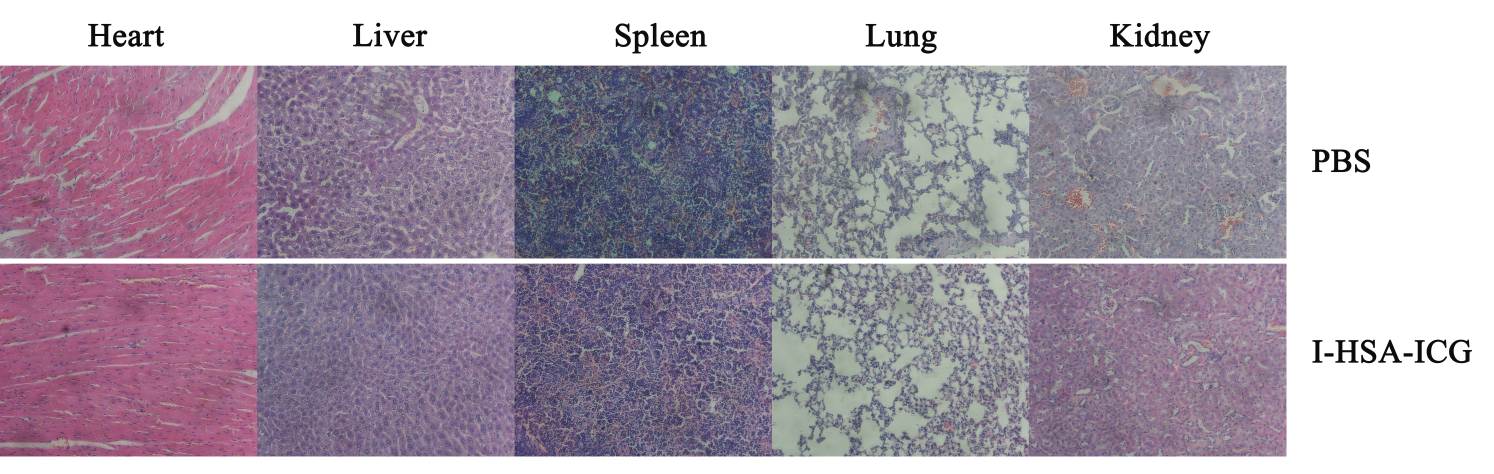
**

**Supplementary Figure 6.** PBS and I-HSA-ICG nanoparticles were injected into the tail vein of two groups of mice, respectively. After 30 days of observation, the heart, liver, spleen, lung and kidney of mice were taken for H&E staining.


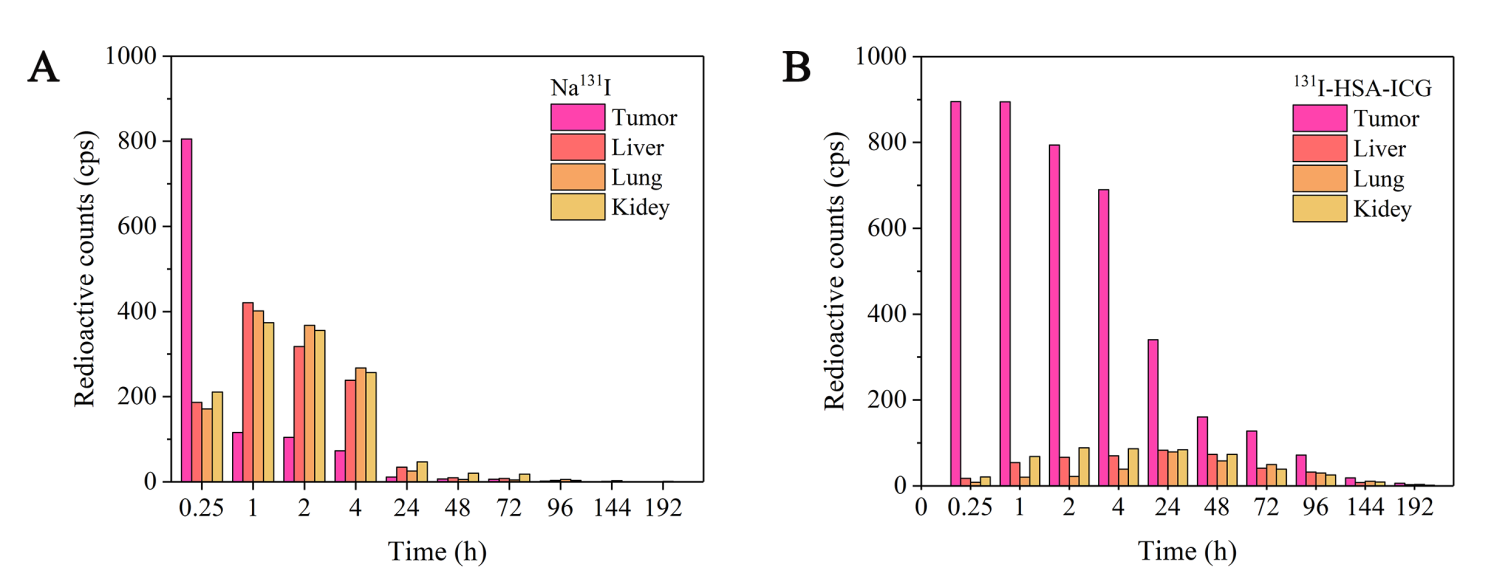


**Supplementary Figure 7.** (A) The radioactive counts distribution of Na^131^I in tumor tissue and various organs at different time points. (B) The radioactive counts distribution of ^131^I-HSA-ICG nanoparticles in tumor tissue and various organs at different time points.


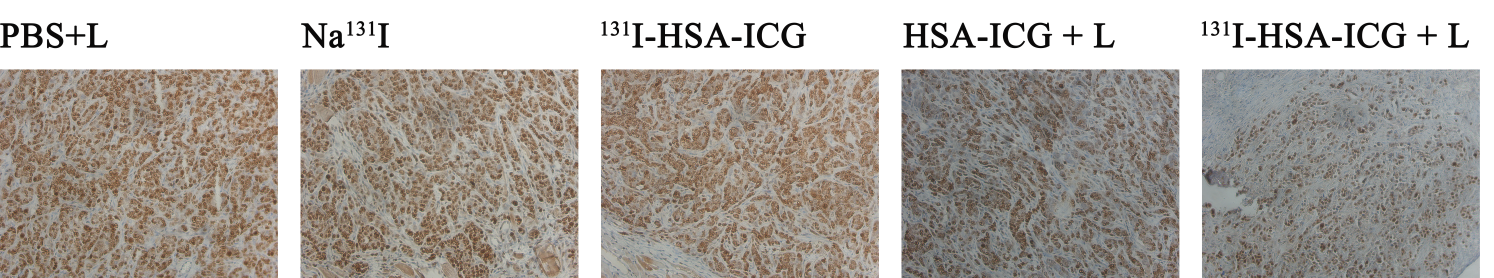


**Supplementary Figure 8.** Ki67 antibody immunohistochemical staining was performed on the treated tumors in each group.


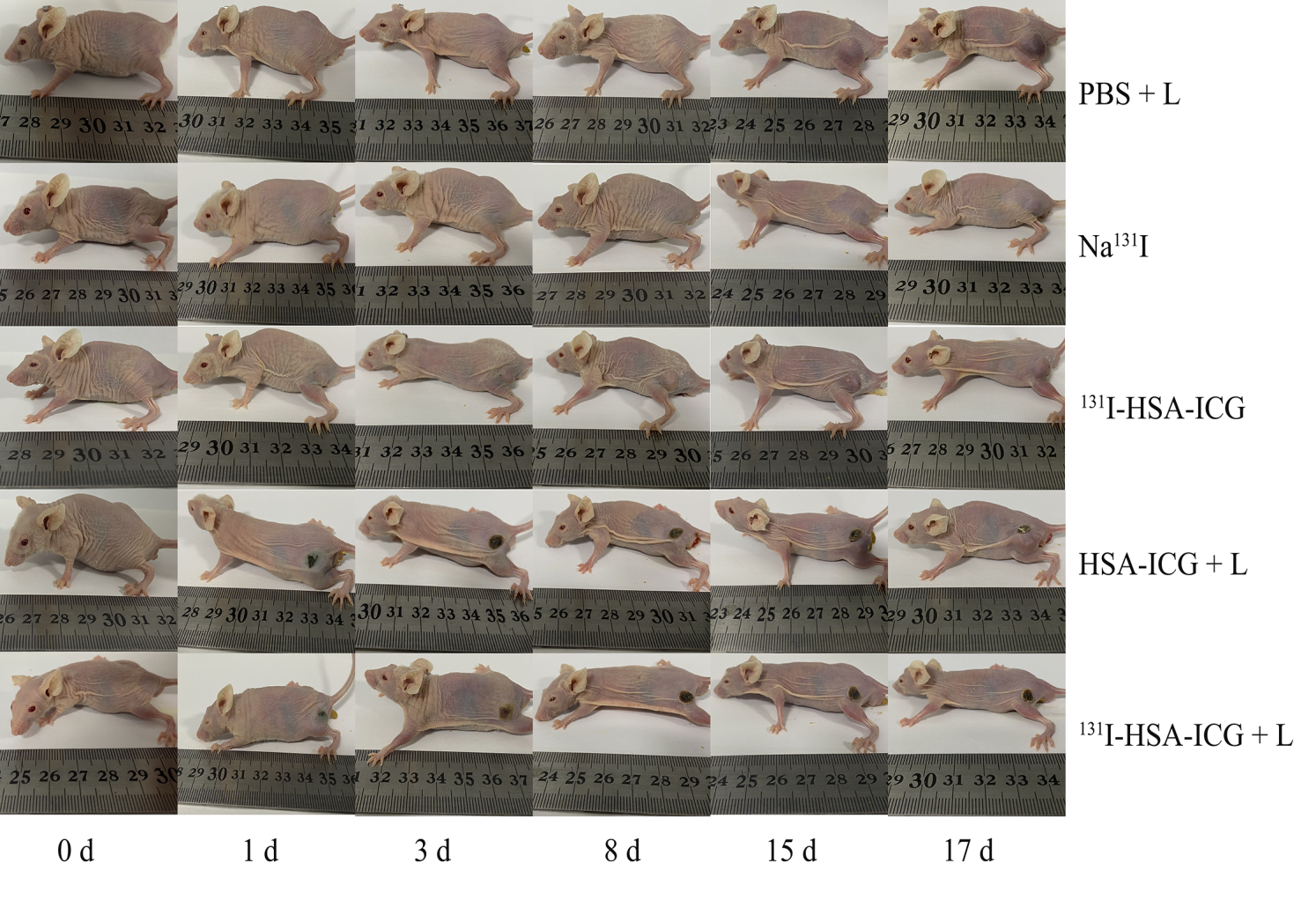


**Supplementary Figure 9.** Nude mice were observed and photographed for 17 days after treatment.





**Supplementary Figure 10.** The trends of body weight changes in nude mice in different treatment groups, expressed as mean ± standard deviation; *, *p <0.05*.

## Supplementary Tables

**Supplementary Table 1** Zeta potential of HSA-ICG/I-HSA-ICG nanoparticles

| **Concentrations (mg/mL)** | **HSA-ICG** | | **I-HSA-ICG** | |
| --- | --- | --- | --- | --- |
|  | **PB** | **H_2_O** | **PB** | **H_2_O** |
| 0.1 | -4.56 | -12.8 | -3.13 | -18 |
| 0.3 | -2.89 | -17.2 | -0.47 | -11.6 |
| 0.5 | -3.06 | -15.6 | -3.18 | -22.2 |
| *PB* Phosphate buffer (pH = 7.4); *H_2_O*, high purity water. | | | | |

**Supplementary Table 2** Effects of I-HSA-ICG nanoparticles on biochemical functions of living body

| **GROUPS (n = 5)** | **PBS** | **I-HSA-ICG** | ***P*** |
| --- | --- | --- | --- |
| ALT | 0.36 ± 0.016 | 0.34 ± 0.023 | 0.150 |
| AST | 26.91 ± 0.610 | 26.39 ± 1.163 | 0.400 |
| GGT | 3.37 ± 0.098 | 3.24 ± 0.112 | 0.087 |
| UREA | 2.56± 0.117 | 2.46 ± 0.244 | 0.433 |
| UA | 36.00 ± 0.707 | 37.40 ± 1.342 | 0.073 |
| CREA | 27.91 ± 0.631 | 27.22 ± 0.686 | 0.134 |
| *PBS* Phosphate buffer saline, *ALT* Alanine aminotransferase, *AST* Aspartate aminotransferase, *GGT* γ-glutamyl transpeptadase, *UA* Uric acid, *CREA* Creatinine. *P* < 0.05 is statistically significant (analyzed by independent-samples T test). | | | |
